# Disorganized behavior on Link's cube test is sensitive to right hemispheric frontal lobe damage in stroke patients

**DOI:** 10.3389/fnhum.2014.00079

**Published:** 2014-02-17

**Authors:** Bruno Kopp, Nina Rösser, Sandra Tabeling, Hans Jörg Stürenburg, Bianca de Haan, Hans-Otto Karnath, Karl Wessel

**Affiliations:** ^1^Department of Neurology, Hannover Medical SchoolHannover, Germany; ^2^Cognitive Neurology, Technische Universität BraunschweigBraunschweig, Germany; ^3^Department of Neurology, Braunschweig HospitalBraunschweig, Germany; ^4^Department of Neurology, Klinik NiedersachsenBad Nenndorf, Germany; ^5^Division of Neuropsychology, Center of Neurology, Hertie-Institute for Clinical Brain Research, University of TübingenTübingen, Germany; ^6^Department of Psychology, University of South CarolinaColumbia, SC, USA

**Keywords:** executive function, problem solving, spatial behavior, Link's cube test, right hemisphere damage, frontal lobe

## Abstract

One of Luria's favorite neuropsychological tasks for challenging frontal lobe functions was Link's cube test (*LCT*). The *LCT* is a cube construction task in which the subject must assemble 27 small cubes into one large cube in such a manner that only the painted surfaces of the small cubes are visible. We computed two new *LCT* composite scores, the constructive plan composite score, reflecting the capability to envisage a cubical-shaped volume, and the behavioral (dis-) organization composite score, reflecting the goal-directedness of cube construction. Voxel-based lesion-behavior mapping (VLBM) was used to test the relationship between performance on the *LCT* and brain injury in a sample of stroke patients with right hemisphere damage (*N* = 32), concentrated in the frontal lobe. We observed a relationship between the measure of behavioral (dis-) organization on the *LCT* and right frontal lesions. Further work in a larger sample, including left frontal lobe damage and with more power to detect effects of right posterior brain injury, is necessary to determine whether this observation is specific for right frontal lesions.

## Introduction

Luria (1966) proposed that the frontal lobes are essential for organizing goal-directed behavioral sequences, and accordingly, that frontal lobe damage disrupts the self-regulated structure of behavior. For frontal lobe patients, individual fragments of sensation and perception, of thought and action may be preserved; yet, the process of organizing these fragments into a useful structure is severely impaired. According to Luria (1966), the plan of action, if existent, loses its regulatory influence on behavior, and the goal-directed structure of behavior is replaced by disorganized behavior. Thus, rather than examining relevant properties and conditions, these patients often behave in an impulsive manner, i.e., without an analysis of what needs to be done, or of what objects and operations are available to do it.

**Figure 1:**
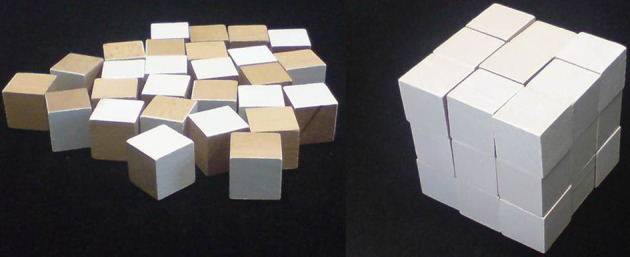
**The stimulus materials of the *LCT* (Metzler, 2000).** Left panel: 27 small wooden cubes (3 by 3 by 3 cm each) are scattered on the table in front of the subject. Eight of them have three white surfaces and three wooden surfaces. Twelve cubes have two white and four wooden surfaces, six cubes have one white and five wooden surfaces. One cube is all wooden. Right panel: The subject is instructed to construct a single large cube—by using all small cubes—whose outer surface is entirely white. Reproduced from Kopp et al. (2008) with permission of the copyright owner.

One of Luria's favorite neuropsychological tasks for challenging frontal lobe functions was Link's cube test (*LCT*; Link, 1919, 1923)^1^. The *LCT* asks patients to construct a single large cube by assembling 27 small cubes in such a manner that only the painted surfaces of the small cubes are visible (see Figure 1). The large cube was first of all presented to the subject in the original *LCT*. Only after its demolition, the subject started to construct a replication, rendering the *LCT* basically a technique for the assessment of visuo-constructive abilities (Link, 1919). In Luria's variant of the *LCT*, the initial presentation of the large cube was omitted^2^, rendering the *LCT* a spatial problem solving task—the problem being defined by the mismatch between the initial state of the scattered small cubes (see Figure 1, left panel) and the spatially arranged final goal state of these cubes (see Figure 1, right panel).

Metzler (2000) published an assessment instrument which he called *Standardisierte Link'sche Probe* (*Standardized Link's Test*). Metzler's variant of Luria's *LCT* introduced 10 behavior ratings to be made by the examiner for various aspects of the examinee's performance^3^. Metzler (2000) also described the results that he obtained from a normative sample (*N* = 220; age in years: *M* = 33.5, *SD* = 14.3; age range 14–60) of healthy individuals and from two patient samples: 69 patients with frontal damage of mixed etiology (age in years: *M* = 38.8, *SD* = 15.0) as well as 38 neurological (without known frontal lobe damage) and psychiatric patients (age in years: *M* = 32.0, *SD* = 12.8). Analyses of the completed ratings for the total sample (*N* = 327) indicated that these measures were highly inter-correlated, with the average correlation amounting to 0.59. A factor analysis identified only one factor that explained over 63 percent of the variance for all 10 measures. Estimates of internal consistency of the 10 scores yielded good coefficients for the total sample (α = 0.93). Consequently, Metzler (2000) focused on the *LCT* global composite measure which he obtained by summing up the 10 rating scores. Estimates of inter-rater reliability of *LCT* scores (correlation coefficients) were reasonably high (*LCT* global composite *r* = 0.98, 0.64 ≤ r ≤ 1.00 for *LCT* scores).

Criterion validity of the *LCT* global composite score could also be established. Metzler (2000) found the *LCT* global composite score to be the neuropsychological measure most sensitive to the presence of frontal lobe damage in the patient group with documented lesions (*N* = 69; the mean *z*-score of these patients amounted to *M_z_* = −2.65; *SD_z_* = 1.56), although the test battery included many tests that are traditionally considered to be tests of frontal functioning (i.e., verbal letter fluency Henry and Crawford, 2004, Stroop test Perret, 1974; Stuss et al., 2001; Demakis, 2004, and Wisconsin Card Sorting Test Milner, 1963; Heaton et al., 1993; Demakis, 2003). Furthermore, the *LCT* global composite score was sensitive to the presence of lateralized frontal lobe damage: Patients with lesions restricted to the right-sided frontal lobe achieved lower global composite *LCT* scores (*M* = 5.6, *SD* = 4.6; in relation to *M* = 21.9, *SD* = 4.8 that were obtained from the *N* = 220 healthy individuals) compared to patients with lesions restricted to the left-sided frontal lobe (*M* = 12.3, *SD* = 8.0). Yet, despite these promising results, more research is needed on the sensitivity of the *LCT* toward (right-lateralized) frontal lobe damage (Kopp et al., 2008).

The study by Metzler (2000) was the first, and to our knowledge, only study suggesting an association between frontal lobe damage and poor performance on the *LCT*. This study, however, assessed patients with lesions from many different etiologies. Specifically, the majority (67%) of his patients suffered from traumatic brain injury. Traumatic brain injury typically results in diffuse and multifocal brain damage that is difficult to visualize and demarcate (Smith et al., 2003) with the full neurological damage extending considerably beyond the borders of the visible lesion. As a consequence, the cognitive deficits displayed by the majority of the patients in the study by Metzler (2000) may not be solely due to the visible lesion in the right frontal lobe.

In the present study, we investigated the sensitivity of performance on the *LCT* (Metzler, 2000) to frontal lobe damage in stroke patients based on a voxel-based lesion-behavior mapping approach (VLBM; Rorden and Karnath, 2004; Rorden et al., 2007, 2009). The VLBM method can be used when the regions of brain injury are sufficiently clearly defined. In this case, the lesions are manually identified for each patient before individual brains are realigned into a common stereotaxic space [i.e., the Montreal Neurological Institute (MNI) space]. Finally, statistical techniques can be applied which test, on the basis of individual brain voxels, whether lesioned voxels are reliably associated with impaired behavior (see Materials and Methods for details).

Metzler's study (2000) suggested sensitivity of performance on the *LCT* to lesions in the frontal lobes, with right frontal damage associated with particularly severe impairment. Here, we are looking to replicate that right frontal association in a sample of stroke patients where lesions are clearly demarcated. Thus, we aimed to replicate and more rigorously assess the hypothesized association between right frontal lobe damage and performance on the *LCT*.

## Materials and methods

### Subjects

Thirty-two (19 male, 13 female) acute first-ever, right-hemisphere-damaged stroke patients with damage centering on or involving the frontal lobe in most patients participated in the study (see Table 1 for details). We solely included right-hemisphere-damaged stroke patients in order to examine whether performance on the *LCT* is sensitive to focal right frontal lesions. In addition, task performance of left-hemisphere-damaged stroke patients might be distorted due to paresis and/or apraxia of the dominant hand. Further, left-hemisphere strokes might have hampered the capability to understand task instructions, due to the potential presence of sensory aphasia^4^. Patients with diffuse or bilateral brain lesions due to traumatic brain injury, brain tumors, subcortical arteriosclerotic encephalopathy, or any other dementing disease were excluded. Patients without prior psychiatric disease or those without alcohol or drug abuse were recruited. Further, patients with gross neurological defects (pronounced pain as reported by the patient, left homonymous hemianopia as revealed by clinical examination, hemispatial visual neglect) were also excluded to make sure that these symptoms did not interfere with task performance^4^. Spatial neglect was diagnosed when a patient showed the characteristic clinical behavior such as orienting toward the ipsilesional side when addressed from the front or the left and/or ignoring contralesionally located people or objects. All patients gave their informed written consent to participate in the study, in accordance with the ethical standards of the Declaration of Helsinki. Table 1 shows demographic and neuropsychological participant characteristics. Appropriate ethical approval was obtained from the Ethics Committee at the Technische Universität Braunschweig.

**Table 1:** **Demographic and neuropsychological patient characteristics**.

	***N***	***M***	***SD***
Age	32	59.66	10.18
Years of education	32	12.27	2.23
Handedness	32	0.94	0.26
ADS-L [*z*]	23	0.06	0.86
MMSE [*RS*]	32	27.47	2.24
WST [*z*]	29	−0.32	0.84
RWT—subtest s-words [*PR*]	32	36.53	25.59
RWT—subtest animals [*PR*]	32	37.88	29.96
MCST—N categories [*RS*]	27	5.30	1.35
MCST—N perseveration errors [*RS*]	27	2.30	3.42

Allgemeine Depressions-Skala, Langform [General depression scale, long form] (ADS-L; Hautzinger and Bailer, 1992; German version of the Center for Epidemiologic Studies Depression Scale, CES-D; Radloff, 1979); handedness, handedness ratio on the Edinburgh Handedness Questionnaire (Oldfield, 1971; −1 = strongly left-handed, 0 = ambidextrous, 1 = strongly right-handed); Mini-Mental-State-Examination (MMSE; Folstein et al., 1983); Modified Card Sorting Test (MCST; Nelson, 1976); Regensburger Wortflüssigkeits-Test [Regensburger word fluency test] (RWT; Aschenbrenner et al., 2000); Wortschatz-Test [vocabulary test] (WST; Schmidt and Metzler, 1992).

Sex: m, male; f, female; years of education: school and vocational education; N, number of patients; PR, percentile rank; M, Mean; RS, Raw Score; SD, Standard deviation; z, z-score.

### Test description, administration and scoring

The stimulus materials and the specifications for administering the *LCT* are presented in Figure 1. The *LCT* behavior ratings consist of the following 10 scores (cf. Metzler, 2000, for more details):

1. **Exploration.** The item provides a rating of behavioral evidence for “preliminary investigative activity,” problem identification, and means-end analysis, most notably inspection and sorting of the small cubes. If patients sorted all cubes, the score was three; if they sorted a subset of cubes, the score was two; if they merely inspected individual cubes, the score was one; if they did not explore the cubes at all, the score was zero.
2. **Spatial sub-goaling.** The item provides a rating about how the edge length of the large cube was planned. If patients counted the small cubes and correctly calculated the edge length of the large cube, the score was three; if they counted, but calculated incorrectly, the score was two; if they counted, but did not calculate, the score was one; if they did not count nor calculate, the score was zero.
3. **Action organization.** The item provides a rating of the capability to organize goal-directed sequences of actions. If patients showed planned, consequential and goal-directed use of the small cubes, the score was three; if they did so, but also showed spontaneous and needless behaviors, the score was two; if they showed grossly disorganized behavior, the score was one; if they showed behavioral chaos, the score was zero.
4. **Mental spatial structure.** The item provides a rating of whether patients built the idea of a three-dimensional cube resting on a quadratic basic shape, and whether their actions followed this anticipated final goal state in a stringent manner. If patients showed behavioral evidence for a three-dimensional imagination, for a quadratic shape, and for a stringent use of the small cubes, the score was three; if they showed behavioral evidence for a three-dimensional imagination, yet placed the small cubes in a highly insecure manner, the score was two; if they failed to show behavioral evidence for both, a three-dimensional imagination and for a quadratic basic shape, the score was one; if they constructed non-quadratic shapes (such as rectangles, rings, or walls) in a single layer, the score was zero.
5. **Attention control.** The item provides a rating of the capability to maintain attention to the color of the outer surface of the large cube. If patients committed only a few surface color errors, and if they corrected these errors during construction, the score was three; if they committed several surface color errors, and if they failed to correct one or two of these errors during construction, the score was two; if they failed to control for surface color errors, and if they failed to correct several of these errors during construction, the score was one; if they committed many surface color errors, and if they failed to correct many of these errors, the score was zero.
6. **Error correction.** The item provides a rating of the organization of error correction, ranging from the goal-directed search for errors by re-constructing specific parts of the cube to the repeated demolishing of the entire cube. If no error correction was required, the score was three; if the search for errors proceeded in a well-regulated manner, the score was two; if the search for errors proceeded in a less orderly manner with some needless cube deconstructions, the score was one; if the search for errors proceeded in a disordered manner with many needless cube deconstructions, the score was zero.
7. **Edge length.** The item provides a rating about how the edge length of the large cube was achieved. If patients reached at the correct edge length of the large cube immediately, the score was three; if they began initially with an incorrect edge length, but corrected the edge length by themselves, the score was two; if they began initially with an incorrect edge length, and if they corrected the edge length only when an obvious lack of small cubes enforced them to do this, the score was one; if they repeatedly constructed their large cube with an incorrect edge length, and if they did not achieve to correct the edge length by themselves, the score was zero.
8. **Final state.** The item provides a rating about the appropriateness of the final state. If the final state was without any error, the score was three; if the final state featured one or two errors, the score was two; if the final state manifested three or five errors, the score was one; if the final state showed many errors, or if the large cube was incomplete, or if the task was aborted, the score was zero.
9. **Number of cues.** The item provides a rating of the number of cues that were given to the patient. If no cues were provided, the score was three; if the instruction was repeated or explained once, the score was two; if the instruction was repeated or explained twice, or if cues on incorrect construction were given, the score was one; if multiple cues were provided, the score was zero.
10. **Time requirement.** The item categorizes the amount of time required on the task. If patients needed less than 4 min, the score was three; if they needed 4–6 min, the score was two; if they needed 6–10 min, the score was one; if they needed more than 10 min, the score was zero.

Instructions were worded as follows: “Your task is to construct one large cube by assembling the many small cubes that lie in front of you. If we look at it, the large cube must appear white throughout. That's why some, but not all, of the surface areas of the small cubes are white. Please bear in mind that the later invisible surface area of the large cube must also be white. None of the small cubes may be left over. And also keep in mind: Cubes are defined as having three sides of equal length. I measure the time it takes you to construct the large cube, but the time it takes is of only negligible importance to me. I am mainly interested in seeing how you solve your task. Do you have questions before we start?”

It is appropriate to provide cues to the patient under the following conditions: (1) The patient constructs walls or rectangles, but not a cube, even after several attempts. (2) The patient uses repeatedly wrong edge lengths. (3) The patient refuses further participation. (4) The patient accomplished the cube, but with errors that are not recognized by the patient. Cues may consist of parts of the instructions, including the explanation of how cubes are defined, or hints on errors.

The *LCT* global composite score was computed (range: 0–30) by summing up the 10 individual *LCT* rating values (Metzler, 2000). Apart from the *LCT* global composite score, two new *LCT* composite scores were computed: First, the *LCT* constructive plan composite score (range: 0–9) comprised the sum composed of (2) spatial sub-goaling, (4) mental spatial structure, and (7) edge length. These three ratings target the capability to mentally form an appropriate constructive plan and to enable this plan to provide a regulatory influence on construction behavior. In Metzler's study (2000), the average inter-correlation between these three rating values amounted to 0.71, a finding that can be considered as evidence for the relatively distinct homogeneity of these three ratings. Second, the *LCT* behavioral (dis-) organization composite score (range: 0–9) comprised the sum composed of (3) action organization, (5) attention control, and (6) error correction. These three ratings target the goal-directedness of single units of behavior with regard to the demand to solve the *LCT* problem efficiently. In Metzler's study (2000), the average inter-correlation between these three rating values amounted to 0.69, a finding that can be considered as evidence for the relatively distinct homogeneity of these three ratings. Note that these averaged intra-score correlations (i.e., 0.71 and 0.69, respectively), should be contrasted with the average inter-correlation between all remaining items after exclusion of these six intra-score correlations, which amounted to 0.57.

There is also a psychometric rationale for combining individual scores to linearly combined composite scores, such as the *LCT* constructive plan composite score and the *LCT* behavioral (dis-) organization composite score. Specifically, the reliability of linearly combined composite measures exceeds the reliabilities of the individual measures upon which they are based (Nunnally and Bernstein, 1994), thereby enhancing the chance to detect brain-behavior relationships.

### Lesion analysis

Magnetic resonance imaging (MRI) was performed in 28 stroke patients and computed tomography (spiral CT) scanning was performed in four patients. The initial scanning was optionally repeated during the following days until the infarcted area became clearly demarcated. The mean time interval between lesion onset and the MRI scan that was used for the present analysis amounted to 4.3 days (*SD* = 3.1); the mean time interval between time of lesion and CT scanning lasted 0.25 days (*SD* = 0.5). MRI scans were obtained on a 1.5 T echo planar imaging (EPI) capable system (Philips Intera, Philips Medical Systems, Best, The Netherlands). The MRI protocol used diffusion-weighted imaging (DWI, *N* = 12) and T2-weighted fluid-attenuated inversion-recovery imaging (FLAIR, *N* = 16). DWI was performed with a single-shot EPI spin echo sequence [25 axial slices; repetition times (TR), either 3690, 4000, 4452, 5060, 5300, or 6360 ms; echo times (TE), either 90, 95, or 120 ms; field of view (FOV), 230 × 230 mm^2^; matrix 64 × 64 pixels; slice thickness, 5 mm; gap, 5.5 mm]. The FLAIR sequences were acquired with 25 axial slices (thickness, 5 mm) with an interslice gap of 5.5 mm, a FOV of 220 × 220 mm^2^, TR of either 4000, 5397, 5500, or 6000 ms, and TE of either 89, 91, 100, or 120 ms. CTs were obtained on a spiral scanning system (Somatom Sensation 16, Siemens Healthcare, Erlangen, Germany) with a slice thickness of 3 mm infratentorial and 6 mm supratentorial and an in-plane resolution of 0.5 × 0.5 mm.

Lesion location was evaluated using *MRIcroN* software (Rorden et al., 2007, www.mricro.com). For patients with MRI scans, the boundaries of lesions were delineated directly on the individual MRI scans. Both the MRI scan and the lesion shape were then mapped into stereotaxic space using the normalization algorithm provided by SPM5 (www.fil.ion.ucl.ac.uk/spm/software/spm5/). Cost–function masking was employed (Brett et al., 2001) for determination of the transformation parameters.

In patients with spiral CT scans, lesions were drawn directly by an experienced neurologist (Hans-Otto Karnath; blinded for test performance) on the slices of a normalized T_1_-weighted template MRI scan from the MNI with a 1 × 1 mm in-plane resolution, distributed with the *MRIcroN* toolset. Lesions were mapped onto the slices that correspond to MNI Z-coordinates [−16, −8, 0, 8, 16, 24, 32, and 40 mm] by using the identical or the closest matching axial slices of each individual patient.

To evaluate the relationship between lesion location and performance on the three *LCT* composite scores [global composite score, constructive plan composite score, and behavioral (dis-) organization composite score], three voxel-based lesion-behavior analyses were performed using the *MRIcroN* toolset (Rorden et al., 2007; www.mricro.com). These statistical analyses were based on the Brunner–Munzel (*BM*) test (Brunner and Munzel, 2000) where, for each voxel, the behavioral scores of patients with a lesion in that voxel and patients with a lesion elsewhere are statistically compared using non-parametrical statistics. Only voxels that were damaged in at least three patients were included in the analysis (*N* = 150497 voxels). We controlled for multiple comparisons using permutation-based thresholding (Kimberg et al., 2007) using 4000 iterations. Significant results presented survived a 5% permutation based false positive probability threshold. We additionally used the *MRIcroN* toolset to calculate power maps originally described for situations where behavioral data is binomial by Rudrauf et al. (2008), and extended to situations where behavioral data is continuous as mentioned by Gläscher et al. (2009), for each of the three voxel-based lesion-behavior analyses. These power maps highlight the areas of the brain where we had enough power to potentially detect a significant effect using the same threshold as our main analyses (*p* < 0.05 using permutation-based thresholding to correct for multiple comparisons).

## Results

### Neuropsychological test results on the *LCT*

Table 2 summarizes the performance of the patients on the *LCT*. The average *LCT* global composite score amounted to *M* = 10.97 (*SD* = 8.65), against *M* = 22.8 (*SD* = 4.3) in healthy males and *M* = 20.8 (*SD* = 5.2) in healthy females of Metzler's (2000) study. Our patients thus, achieved higher *LCT* global composite scores than Metzler's (2000) right frontal lesion patients (*M* = 5.6, *SD* = 4.6), but slightly lower *LCT* global composite scores than Metzler's (2000) left frontal lesion patients (*M* = 12.3, *SD* = 8.0). Task difficulty differed between the 10 *LCT* ratings, with *LCT* mental spatial structure being the easiest item (*M* = 1.72, *SD* = 0.96), and *LCT* time requirement being the most difficult item (*M* = 0.66, *SD* = 0.83). The average *LCT* constructive plan composite score amounted to *M* = 4.28 (*SD* = 3.12), against *M* = 6.24 (*SD* not provided) in the total sample (*N* = 327; i.e., healthy controls and patients) of Metzler (2000). The average *LCT* behavioral (dis-) organization composite score amounted to *M* = 3.00 (*SD* = 2.74), against *M* = 5.34 (*SD* not provided) in the total sample (*N* = 327; i.e., healthy controls and patients) of Metzler (2000).

**Table 2:** **Neuropsychological results on the *LCT***.

***LCT* score**	***M***	***SD***	**Mdn**	**IQR**
Global composite	10.97	8.65	8.00	11.50
Exploration	0.84	0.99	1.00	1.00
Spatial sub-goaling	1.28	1.11	1.00	2.00
Action organization	1.16	1.01	1.00	2.00
Mental spatial structure	1.72	0.96	2.00	1.00
Attention control	0.97	1.00	1.00	2.00
Error correction	0.88	0.98	1.00	1.00
Edge length	1.28	1.37	1.00	3.00
Final state	1.19	1.12	1.00	2.00
Number of cues	1.00	1.14	1.00	1.75
Time requirement	0.66	0.83	0.00	1.00
Constructive plan composite (2 + 4 + 7)	4.28	3.12	3.00	5.75
Behavioral (dis-) organization composite (3 + 5 + 6)	3.00	2.74	3.00	4.75

IQR, inter-quartile range (Q_75_–Q_25_).

### Lesion analyses: *LCT* scores

Table 3 summarizes the results obtained with the BM-test over the three *LCT* composite scores [*LCT* global composite score, *LCT* constructive plan composite score, and *LCT* behavioral (dis-) organization composite score] to identify whether or not there were voxels that, when injured, were associated with the presence of behavioral disturbances on the *LCT*. Statistical significance was found solely for the behavioral (dis-) organization composite score (i.e., the sum over the items 3, 5, and 6).

**Table 3:** **Brunner–Munzel test statistics (maximum Brunner–Munzel *z*-score, critical Brunner–Munzel *z*-score) over the three *LCT* composite scores**.

***LCT* score**	**max. *BMz***	***z*_crit_**
Global composite	3.121	3.390
Constructive plan composite (2 + 4 + 7)	3.121	3.481
Behavioral (dis-) organization composite (3 + 5 + 6)	3.320^*^	3.239

* p < 0.05.

### Lesion analyses: lesion overlap and power maps

**Figure 2:**
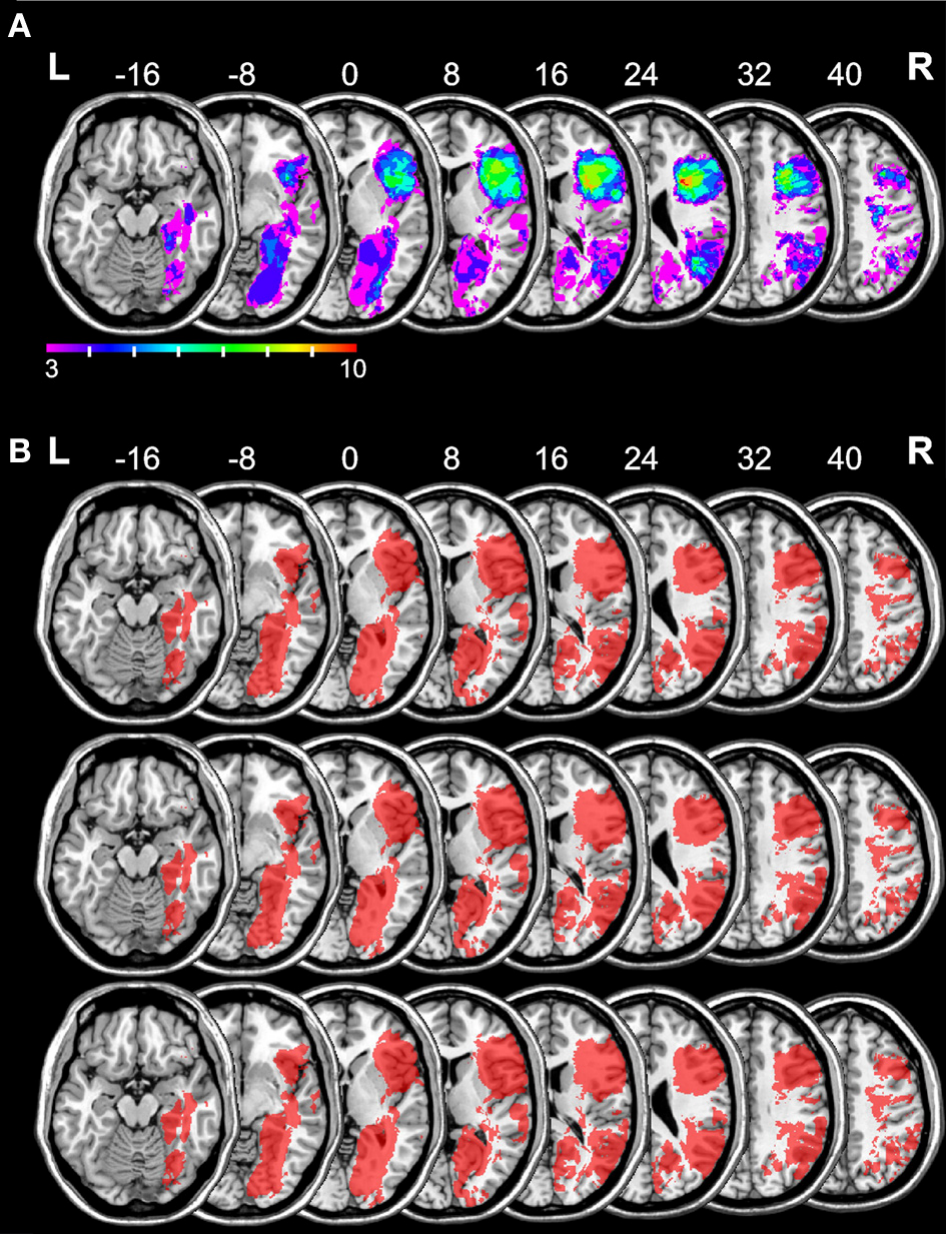
**Overlay lesion plot of all 32 patients and the results of the retrospective power analyses for each of the three *LCT* composite scores.** The number of overlapping lesions **(A)** is illustrated by color, from violet (*N* = 3) to red (*N* = 10). Maximum overlap occurred in the right frontal lobe. The results of the power analyses **(B)** are shown in red [top row: *LCT* global composite, middle row: *LCT* constructive plan composite, bottom row: *LCT* behavioral (dis) organization composite]. These power maps demonstrate that in all areas where the lesions of at least three patients overlapped, we had sufficient power to potentially detect a significant difference between the behavioral scores of patients with a lesion and the behavioral scores of patients without a lesion. Numbers indicate MNI z-coordinates.

Figure 2A shows an overlay lesion plot of all 32 patients in eight axial slices of a standard brain (i.e., in MNI space). Inspection of Figure 2A reveals that the maximum lesion overlap occurred in the right prefrontal cortex (PFC) where up to 10 patients showed lesions in single voxels. Figure 2B shows the results of the retrospective power analyses for each of the three *LCT* composite scores. These power maps demonstrate that in all areas where the lesions of at least three patients overlapped, we had sufficient power to potentially detect a significant difference between the behavioral scores of patients with a lesion and the behavioral scores of patients without a lesion.

### Lesion analyses: *LCT* behavioral (dis-) organization composite score

**Figure 3:**
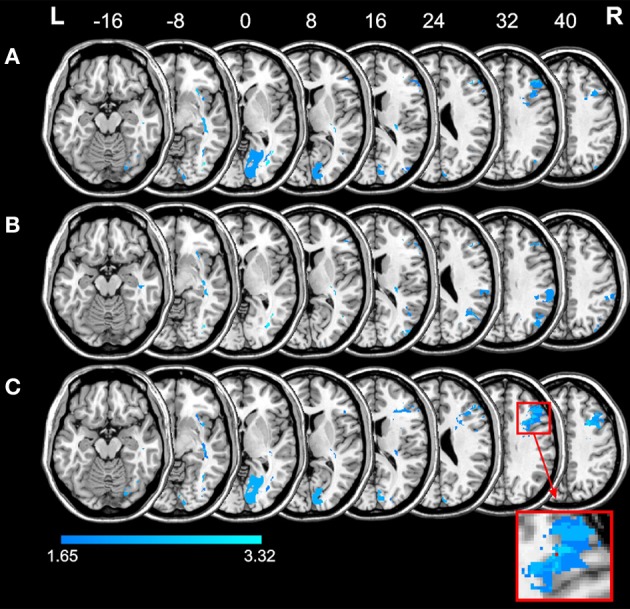
**Anatomical results obtained from the voxel-based lesion-behavior mapping on the *LCT* global composite score (A), the *LCT* constructive plan composite score **(B)**, and the *LCT* (dis-) organization composite score **(C)**.** The anatomical results without control for multiple comparisons (*z*_crit_ = 1.65) are depicted in blue. The significant result obtained for the *LCT* behavioral (dis-) organization composite score is shown in red (see magnified cut-out for a better view). Numbers indicate MNI z-coordinates.

Figure 3C depicts the location of those voxels for which the voxel-based lesion-behavior analysis revealed a significant association between voxel damage and the *LCT* behavioral (dis-) organization composite score (cf. Table 3). Inspection of this map reveals a particular area within the right frontal lobe that is statistically related with low *LCT* behavioral (dis-) organization composite scores. Specifically, a significant *BMz* value of 3.32 was found in a voxel at MNI coordinates *X* = 37, *Y* = 19, *Z* = 32, a white matter coordinate underneath cortical area BA9 (depicted in red, see also the magnified cut-out). The presence of a lesion in this voxel was associated with lower *LCT* behavioral (dis-) organization composite scores.

Figure 3 additionally shows the results of the statistical analysis without correction for multiple comparisons for the *LCT* global composite score, for the *LCT* constructive plan composite score, and for the *LCT* (dis-) organization composite score (blue). These maps allow the reader to assess whether the single significant voxel really represents a statistical value that differs meaningfully from statistical values obtained from other areas of the brain. As can be seen from Figure 3A, the *LCT* global composite score was associated with lesions in two regions of the brain, notably an occipital and a lateral prefrontal region, but none of the voxels within these regions survived correction for multiple comparisons. Further, as revealed by Figure 3B, superior parietal lesions and fronto-parietal white matter lesions led to decrements in the *LCT* constructive plan composite score, but again, none of the voxels within these regions survived correction for multiple comparisons. Finally, Figure 3C reveals an occipital and a lateral prefrontal region related to (dis-) organized composite performance on the *LCT*, but only a single voxel within the lateral prefrontal region (see above) reached the conventional level of significance after correction for multiple comparisons.

## Discussion

We observed a relationship between performance on the *LCT* and frontal lobe injury in a sample of stroke patients with right hemisphere damage. Specifically, as revealed by VLBM, right frontal lesions affected the measure of behavioral (dis-) organization on the *LCT*. The association between frontal lobe damage and behavioral (dis-) organization on the *LCT* surpassed significance in a single voxel within the right frontal lobe (BA9). The current study adds to the literature in multiple ways: First, it represents a shift from a purely clinical approach toward a more scientific one when it is compared to Luria's (1966) approach. Second, whereas Metzler's (2000) sample included patients with traumatic brain injury in its majority, our sample consisted solely of stroke patients with clearly demarcable lesions. Traumatic brain injury results in diffuse and multifocal brain damage, such that cognitive deficits displayed by the vast majority of the patients in the study by Metzler (2000) may not be solely due to their visible frontal lesions. Notwithstanding these shortcomings, Metzler (2000) reported a sensitivity of the *LCT* global composite score to frontal lesions, with right frontal damage associated with particularly severe impairment. Here, we established for the first time an association between the newly developed *LCT* (dis-) organization composite score and focal lesions in the right frontal lobe.

The relationship between right frontal damage and behavioral (dis-) organization on the *LCT* is of importance against the background that there are few measures available for assessing functional disability in right frontal patients (Lezak, 1995; Vallesi, 2012). For example, while verbal fluency can be considered a test of left frontal function (Henry and Crawford, 2004; Baldo et al., 2006), nonverbal analogs of verbal fluency, such as design fluency, do not seem to provide comparably sensitive and specific indices of right frontal function (Baldo et al., 2001). However, the data presented here need to be interpreted with caution mainly for three reasons: First, the majority of the patients in the current sample had right frontal lesions, and an extended sample should include both patients with right and left frontal lobe damage in order to examine whether or not our observation is specific for *right* frontal lesions. Second, the extended sample should also include many more patients with right posterior damage in order to examine whether or not our observation is specific for *frontal* lesions. With regard to this issue it is interesting that the *LCT* constructive plan score was associated with lesions in superior parietal areas of the right cerebral hemisphere. However, this finding needs replication in a sample of stroke patients with right posterior damage since none of the voxels within these parietal regions survived correction for multiple comparisons. Third, exclusion of patients with visual field defects or with hemispatial neglect generally reduces the generalizability of our claims.

According to our clinical experience, the *LCT* is an ingenious method to assess core aspects of executive behavior (Kopp et al., 2008; Kopp, 2012), and according to the results that we obtained in the current study, the *LCT* behavioral (dis-) organization composite score is a promising tool for the assessment of neuropsychological sequelae of right frontal damage. Further, the *LCT* is a non-routine task that requires solving a rather unfamiliar problem (Karnath et al., 1991). Numerous earlier neuropsychological studies addressed problem solving abilities (e.g., Goel and Grafman, 1995; Morris et al., 1997; Carlin et al., 2000; Colvin et al., 2001; Goel et al., 2001; see Grafman, 2007; Goel, 2009, for reviews). Most widely used are Tower Tests (Tower of Hanoi: Glosser and Goodglass, 1990; Tower of London: Shallice, 1982; Tower of Toronto: Saint-Cyr et al., 1988). Goel and Grafman (1995) have argued that while the well-structured Tower Tests are interesting cognitive tasks, they must be considered sub-optimal tasks because problem solving deficits typically emerge in ill-structured real-world situations^5^. As a consequence of this, a number of researchers have moved beyond well-structured neuropsychological tests and tried to approximate real-world situations (Eslinger and Damasio, 1985; Shallice and Burgess, 1991; Dimitrov et al., 1996; Goel et al., 1997; Channon and Crawford, 1999; Goel and Grafman, 2000). With regard to the ill-structured/well-structured distinction, the *LCT* should be considered as an intermediate task that is less well-structured than Tower Tests, but that is also less ill-structured than typical real world tasks. It should also be noted that the *LCT* is a dual task (Baddeley et al., 1997) since efficient performance on it requires patients to divide attention between achieving the cubical-shaped volume and the requested color surface.

To conclude, our findings suggest that aspects of performance, namely the degree of behavioral (dis-) organization, on the *LCT* are sensitive to right frontal lobe damage. However, all our patients suffered from damage to the right frontal lobe and we can thus, not compare the performance of patients with damage to the right frontal lobe to the performance of patients with damage elsewhere. We can, as a consequence, not draw firm conclusions concerning the specificity of the relationship between damage to the right frontal lobe and behavioral (dis-) organization on the *LCT*. Specifically, future work should examine performance on the *LCT* in patients with left frontal lesions and in patients with posterior lesions. Future collection of data should also identify the extent to which behavioral (dis-) organization on the *LCT* maps on real-world behaviors. Finally, we would like to stress that the scoring system is the major weakness of the *LCT* assessment since some of the behavioral measures seem overly subjective. Improving the assessment of performance on the *LCT* might be found in the application of virtual reality techniques to minimize the influence of non-objective factors that potentially affect *LCT* scores. Further improvements of the quantitative scoring system for assessing dysexecutive behavior on the *LCT* will eventually enhance the objectivity, reliability and validity of this assessment technique.

### Conflict of interest statement

The authors declare that the research was conducted in the absence of any commercial or financial relationships that could be construed as a potential conflict of interest.

## Footnotes

^1^Henry Charles Link (1889–1952) was a psychologist and the author of a book which was entitled Employment Psychology—The Application of Scientific Methods to the Selection, Training, and Rating of Employees (Link, 1919). He described the cube test in Chapter XI (entitled Apprentice tool makers and machinists, pp. 123–129) of this book: “The cube test consists of a three-inch cube, painted in green on the outside. The cube is cut into 27 one-inch cubes. The large cube is placed before the subject and he is told that it will be demolished into 27 small cubes and that he must restore them so that the large cube looks exactly as it did before, viz., green on all sides with no wood color exposed.” (p. 124). (cf. www.archive.org/texts/flipbook/flippy.php?id=employmentpsycho031643mbp, downloaded 4-12-2011. This quote follows the second edition of the book which was published in 1924). In a journal article which was published some years later, Link described the cube test in the following words: “The first (… of three tests …) consisted of a three-inch cube, painted black on the outside and then cut up into 27 one-inch cubes. The 27 small cubes were scattered on a table and the apprentice was asked to put them together into one three-inch cube which, like the original, was entirely black with no natural wood surface exposed.” (Link, 1923, p. 37). Interestingly, Link (1919, 1923) did not provide any information on how to score subjects' performance on his cube test.

^2^Luria's variant of the *LCT* seems to be based on Link's (1923) journal article since this description of the task did not mention the initial presentation of the large cube to the subject.

^3^Metzler's variant of the *LCT* was substantially based on his professional experience in Luria's laboratory (Metzler, pers. commun., May 13, 2003). The second edition of the Standardisierte Link'sche Probe is available since 2012 from Hogrefe Publishing, Göttingen, Germany.

^4^A possible statistical solution to the problem would be to use the severity of hemiparesis, apraxia, aphasia, pain, hemianopia, neglect and other neuropsychological disturbances as covariates. However, covariance analysis presupposes the separation of patients into meaningful groups of individuals, as in neuropsychological group studies, and it further requires a number of restrictive conditions to be met such as, for example, that the slopes of the regression lines (which relate covariates and dependent variables), fitted to the groups, to be parallel.

^5^The ill-structured/well-structured distinction classifies problems based on the amount of information that is available to the problem solver (Reitman, 1964). Tower Tests are typical examples of well-structured problems. In such tasks, the start state is completely specified, as is the goal state and the set of legal state transformations. Planning a meal for guests is a paradigmatic example of an ill-structured task. Here, the start state, the goal state, and the transformation function are incompletely specified (Goel, 1995).
